# A scoping study of pulmonary paracoccidioidomycosis: severity classification based on radiographic and tomographic evaluation

**DOI:** 10.1590/1678-9199-JVATITD-2022-0053

**Published:** 2022-11-25

**Authors:** Sergio Marrone Ribeiro, Thiago Franchi Nunes, Ricardo de Souza Cavalcante, Anamaria Mello Miranda Paniago, Beatriz Aparecida Soares Pereira, Rinaldo Poncio Mendes

**Affiliations:** 1 Department of Infectology, Dermatology, Diagnostic Imaging and Radiotherapy, Botucatu Medical School (FMB), São Paulo State University (UNESP), Botucatu, SP, Brazil.; 2 School of Medicine, Federal University of Mato Grosso do Sul (UFMS), Campo Grande, MS, Brazil.; 3 Graduate Program in Infectious and Parasitic Diseases, Federal University of Mato Grosso do Sul (UFMS), Campo Grande, MS, Brazil.

**Keywords:** Paracoccidioidomycosis, *Paracoccidioides* sp, Severity classification, Radiographic evaluation

## Abstract

The lungs have great importance in patients with paracoccidioidomycosis since they are the portal of entry for the infecting fungi, the site of quiescent foci, and one of the most frequently affected organs. Although they have been the subject of many studies with different approaches, the severity classification of the pulmonary involvement, using imaging procedures, has not been carried out yet. This study aimed to classify the active and the residual pulmonary damage using radiographic and tomographic evaluations, according to the area involved and types of lesions.

## Background

Paracoccidioidomycosis (PCM) is a systemic infectious disease caused by fungi of the *Paracoccidioides* genus, which affects mainly rural workers from Latin America and remains a neglected disease [1]. It presents a gamut of clinical manifestations, with two main clinical forms of active disease - the acute/subacute form (AF) and the chronic form (CF). CF is the most prevalent, ranging from 75% to 95% of the cases, depending on the region taken into account. In the CF, pulmonary involvement is the rule, so the exceptions are patients with normal lungs. Conversely, pulmonary involvement is a rare finding in the AF, affecting only about 5% of the cases [2].

The classification of severity should be carried out individually in order to define several points of each patient management, such as hospitalization, general measures, antifungal compound choice, and treatment of aggravating factors, as well as to certify a sick leave. 

After treatment, most of the PCM patients show fibrotic scars and other sequelae in the involved organs, with higher incidence in the lungs. Consequently, as these patients present the residual form, their sequelae should be evaluated regarding its severity, mainly in the lungs, because of the prevalence of their involvement and the impairment of their function, in connection with the patient’s job. The determination of the pulmonary sequelae severity contributes to the evaluation of the patient condition to carry out their former job. Such evaluation can lead to job maintenance, changing jobs, or even retiring, all of which with important social consequences.

Pulmonary involvement in PCM patients has been evaluated according to several aspects - clinical [3-7], radiographic [3-5, 7-9], function tests [10-12] tomographic [6,7,9,12-16], tomographic-pathologic correlation [17], the six-minute walk test [12], health-related quality of life questionnaires [12], residual lesions [8,12,18], and quantification of fibrosis and emphysema [18]. Nevertheless, these studies have not presented a radiographic and/or tomographic severity classification specific for PCM. 

The clinical classification of severity is well-defined [2,19]. However, the degrees of the pulmonary damage using imaging procedures was performed only by Tobón et al. [8], using a classification proposed for the radiographic evaluation of pneumoconiosis, based only on the altered pulmonary area [20]. A systematic review performed in six databases - PubMed, SciELO, LILACS, Web of Science, BVS and Cochrane using the strategy paracoccidioidomycosis AND radiographic or tomographic severity classification until July 2022 did not identify any publication. Thus, to the best of our knowledge, the severity of the paracoccidioidal pulmonary involvement using imaging procedures has not been characterized.

This study aimed to classify the severity of the pulmonary involvement in PCM patients on chest radiograph and chest computed tomography (CT).

## Radiographic classification

**Figure 1.**
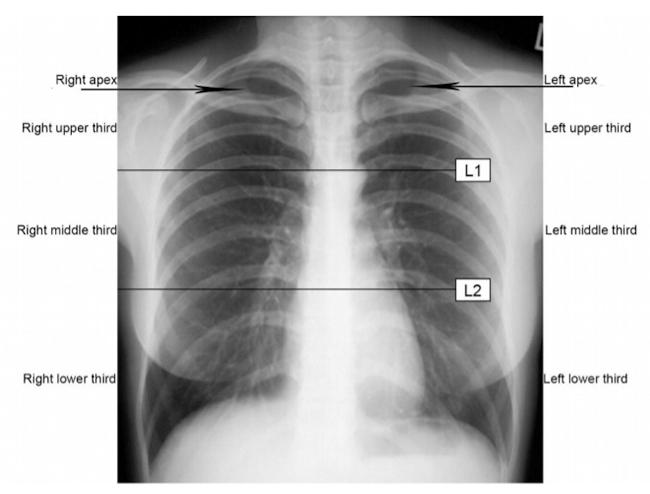
Areas of the chest radiograph.

To classify the severity of the paracoccidioidal pulmonary alterations on radiograph, the lungs should be divided into three parts [21]:

- Upper third: area inferiorly limited by a horizontal imaginary line that passes in the tracheal bifurcation (carina), called line 1 (L1).
- Middle third: area that initiates at L1 and ends in the imaginary line called line 2 (L2), which divides the area limited by L1 and the superior left edge of the diaphragm into two equal parts.
- Lower third: area limited by L2 and the diaphragm (Figure 1).

The pulmonary apices, inferiorly delimited by the upper borders of the clavicles, will be considered as part of the upper thirds, defined above. 

**Figure 2.**
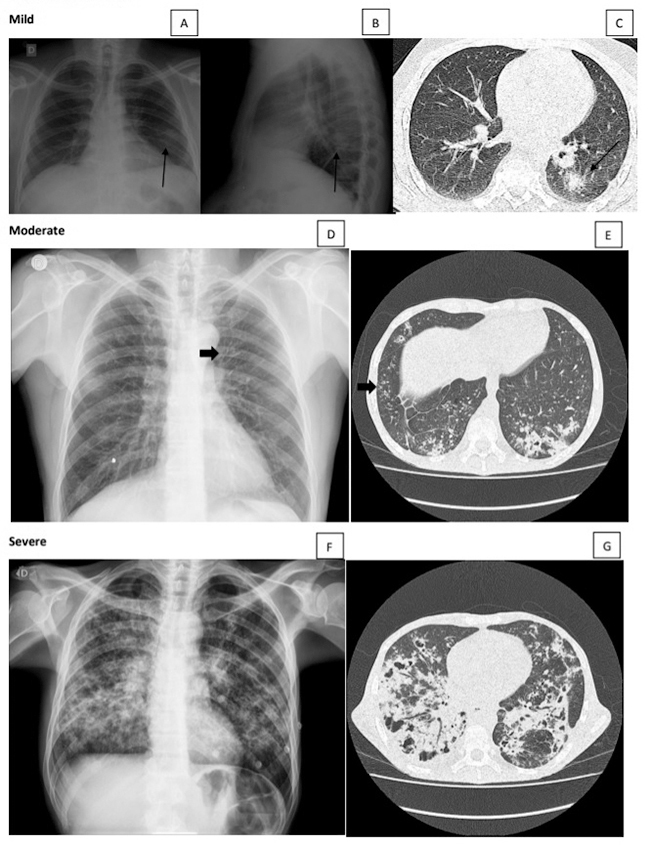
**(A, B, C)** Mild active lesion. Chest radiograph and computed tomography (CT) scan of the same patient showing a solitary pulmonary nodule. Moderate active lesion: **(D)** chest radiograph showing intersticial lesions with bronchial wall thickening (arrow) and **(E)** CT scan showing consolidation, ground-glass opacities, and intersticial lesions with tree-in-bud pattern (arrow). **(F, G)** Severe active lesion. Mixed lesions involving > 1/3 of the total pulmonary parenchyma with cavitary lesions.

**Figure 3.**
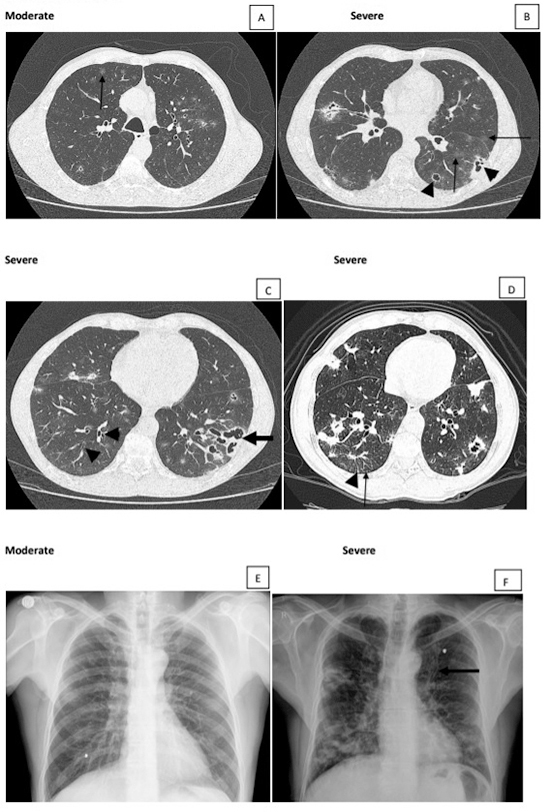
Moderate and severe active lesions. **(A)** Moderate lesion showing > 3 ground-glass nodules (arrow). **(B)** Besides ground-glass nodules (arrows), cavitary lesions with and without septations (arrowheads) in greater amount, characterizing severe classification. **(C)** Bronchiectasis inside consolidation opacity (arrow) and bronchial wall thickening (arrowheads). **(D)** Focal lesions, ground-glass and intersticial opacities with interlobular septal thickening (arrowhead) and small centrilobular nodules (arrow). **(E)** Chest radiograph showing only interstitial opacities, a finding in which CT helps to better characterize and classify the lesions. **(F)** Bronchial wall thickening (tram-track sign) better observed in this patient with severe lesion (arrow).

**Figure 4.**
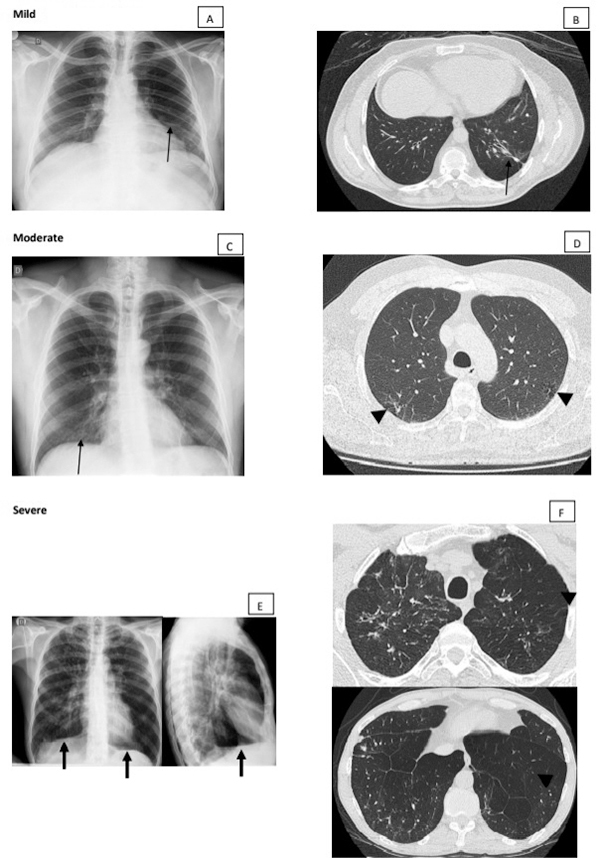
**(A, B)** Mild residual lesion. Chest radiograph and CT scan of the same patient showing parenchymal band associated with traction bronchiectasis on CT (arrows). Moderate residual lesion: **(C)** chest radiograph showing intersticial lesions in subhilar regions (arrow) and **(D)** CT scan showing focal paracicatricial emphysema adjacent to irregular septal thickening (arrowhead). Severe residual lesion: **(E)** chest radiograph showing interstitial fibrotic lesions and signs of pulmonary hyperinflation, such as low set and flattened diaphragm (arrow). **(F)** These emphysema signs are better characterized by CT scan (arrowheads).

**Figure 5.**
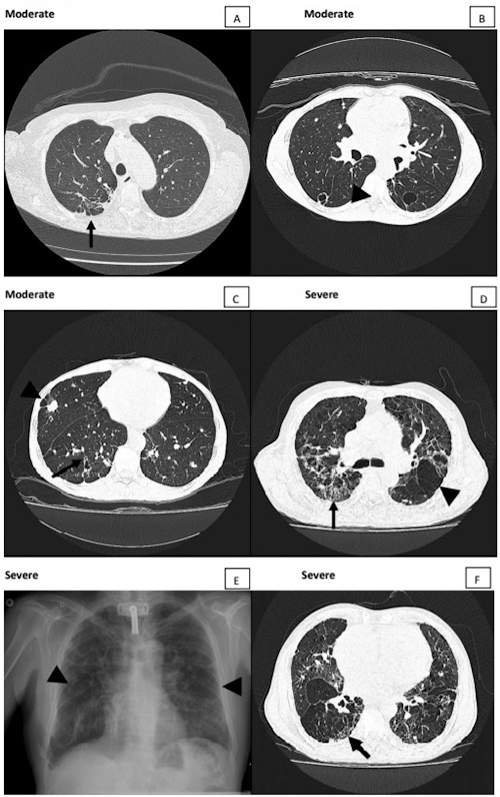
Moderate and severe residual lesions. **(A)** Fibrotic lesions with bronchial wall thickening, traction bronchiectasis, paracicatricial emphysema, and pleural adherences simulating tuberculosis sequalae. **(B)** Moderate residual lesion showing thin wall cavity-gas-filled spaces. **(C)** Paracicatricial emphysema beside nodule (arrowhead) and bronchial wall thickening (arrow) in a moderate residual lesion. Severe residual lesions showing fibrotic lesions with honeycomb lung (arrows in **D** and **F**) and emphysema signs (arrowheads in **D** and **E**).

The pulmonary lesions observed on the chest radiograph of PCM patients with ***active*** disease can be classified as alveolar, interstitial, and mixed. The active alveolar lesions are consolidations simulating snowflakes (Figures 2F and 2G). The interstitial lesions can be reticular, characterized by thin linear opacities (Figures 2D and 4C); nodular, characterized by small (< 1.0 cm in diameter) nodules; or reticulonodular (Figure 4E), when both types of lesions are associated. The mixed lesions present both alveolar and interstitial opacities (Figures 2F and 3A), with predominance of one of them at times. In addition, nodules larger than 1.0 cm in diameter can be observed, and they are called masses when the diameter is larger than 3.0 cm. Cavitary lesions, thickened walls bronchi, and bronchiectasis can be detected (Figures 3B, 5B, 3A, 3C). Thickening or pleural adherences are rarely seen on radiographs, which is different from a computed tomography (CT) scan. Pleural effusion is seldom found, suggesting other etiological diagnosis. 

The ***residual*** lesions shown after efficacious treatment are characterized by the presence of fibrotic scars and/or signs of hyperinflation. The fibrotic scars are manifested as parenchymal bands, coarse nodules, wall thickened bronchi, and thin-walled cavities. The signs of hyperinflation are represented by low and flattened diaphragm, increased retrosternal airspace, hyperlucent areas, and thin-walled bullae. (Figures 4E and 5E).

The active and the residual lesions are classified as mild, moderate, and severe based on the area of the parenchyma involved with alterations and the type of lesions (Table 1). 

**Table 1.** Severity of pulmonary involvement in paracoccidioidomycosis patients on chest radiograph. Evaluation of active disease and residual form.

Severity	Active disease	Residual form
**Mild**	One or two focal lesions^*^ in the same third or not; **or** pure alveolar lesions of any size. *Other possible findings*: nodules.	Discrete fibrotic lesions, mainly parenchymal bands in middle pulmonary third. Normal chest radiograph: rare.
**Moderate**	≥ 3 focal lesions **or** interstitial or mixed lesions involving < ⅓ of the total pulmonary parenchyma. *Other possible findings:* Nodules, bronchial wall thickening (tram-track sign).	Fibrotic lesions: - parenchymal bands; - coarse nodules; - wall thickened bronchi; - thin-walled cavities.
**Severe**	Interstitial or mixed lesions involving > ⅓ of the total pulmonary parenchyma area. *Other possible findings*: nodules, bronchial wall thickening, bronchiectasis, cavitary lesions.	Fibrotic lesions: - parenchymal bands, and/or - coarse nodules, and/or - wall thickened bronchi, and/or - ≤ 2 mm thin-walled cavities; **plus** At least three of the signs of pulmonary hyperinflation below: - low and flattened diaphragm; - increased retrosternal airspace; - hyperlucent areas; - bullae.

*The authors use the expression focal lesions for alveolar opacities in consolidation or ground-glass opacities, nodules, or masses.

## Tomographic classification

### Terms for thoracic imaging

The chest lesions observed on CT scans will be defined according to Fleischner Society [22] and some other authors [23,24], as summarized below.

- Architectural distortion: abnormal displacement of bronchi, vessels, fissure, or septa.
- Atelectasis: reduced inflation of all or part of the lung; collapse is often used interchangeably. On CT scan it is seen as a reduced volume accompanied by increased attenuation in the affected part of the lung.
- Cicatrization atelectasis [23]: a form of lung atelectasis that occurs as a result of scarring or fibrosis that reduces lung expansion. Cicatrization atelectasis is classic in tuberculosis. The term is closely related to cicatrization collapse when an entire lobe is collapsed from the same process.
- Bulla: a rounded focal lucency or area of decreased attenuation, 1 cm or more in diameter, bounded by a thin wall.
- Bronchiectasis: irreversible localized or diffuse bronchial dilatation; it may be classified as cylindric, varicose, or cystic, depending on the appearance of the affected bronchi.
- Traction bronchiectasis and traction bronchiolectasis: respectively represent irregular bronchial and bronchiolar dilatation caused by surrounded retractile pulmonary fibrosis.
- Cavity-gas-filled space: seen as a lucency or low-attenuation area, within pulmonary consolidation, a mass, or a nodule.
- Centrilobular: region of the bronchovascular core of a secondary pulmonary lobule.
- Consolidation: a homogeneous increase in pulmonary parenchymal attenuation that obscures the margins of vessels and airway walls.
- Cyst: any round circumscribed space that is surrounded by an epithelial or fibrous wall of variable thickness. It appears as a round parenchymal lucency or low-attenuating area with a well-defined interface with normal lung. They have variable wall thickness but are usually thin-walled (2 mm).
- Emphysema: permanently enlarged airspaces distal to the terminal bronchiole with destruction of alveolar walls. It is usually classified in terms of the part of the acinus predominantly affected.
- Bullous emphysema: bullous destruction of the lung parenchyma, usually on a background of paraseptal or panacinar emphysema.
- Centrilobular emphysema: characterized by destroyed centrilobular alveolar walls and enlargement of respiratory bronchioles and associated alveoli; on CT scan, centrilobular areas of decreased attenuation, usually without visible walls, of non-uniform distribution and predominantly located in upper lung zones.
- Paracicatricial emphysema [23,24]: dilated terminal air spaces adjacent to a scar in the lung.
- Ground-glass opacity: opacity of the lung, with preservation of bronchial and vascular margins.
- Honeycombing: destroyed and fibrotic lung tissue containing numerous cystic airspaces with thick fibrous walls. On CT scans, the appearance is of clustered cystic air spaces, typically of comparable diameters on the order of 3-10 mm but occasionally as large as 2.5 cm. It is usually subpleural and is characterized by well-defined walls.
- Interlobular septa thickening: sheet-like structures of 10-20 mm long that form the border of lobules. They are more or less perpendicular to the pleura in the periphery. They are not usually seen in the healthy lung, but clearly visible when thickened. On CT scan it may be smooth or nodular.
- Nodule: appears as a rounded or irregular opacity, well or poorly defined, measuring up to 3 cm in diameter.
- Parenchymal band: a linear opacity usually of 1-3 mm thick and up to 5 cm long that usually extends to the visceral pleura.
- Tree-in-bud pattern: represents centrilobular branching structures that resemble a budding tree, which is more pronounced in the lung periphery. The pattern reflects a spectrum of endo- and peribronchiolar disorders, including mucoid impaction, inflammation, and/or fibrosis.

### Lesions observed in PCM patients with active disease

Patients with active PCM present consolidation, ground-glass opacities, or interstitial lesions (Figures 2G, 3B and 2E), involvement of the axial interstitium, with wall-thickened bronchi and centrilobular nodules sometimes with tree-in-bud pattern, and involvement of peripheral interstitium with interlobular septa thickening (Figures 4D, 5A, 2G). Greater nodules and/or masses, with or without cavitation, and bronchiectasis can be observed (Figures 2A, 2B, 2C, 3B, 3C, 5C); however, pleural effusion is rarely observed. 

### Residual lesions

Residual lesions, observed after appropriate treatment, are characterized by signs of fibrosis, such as parenchymal bands, cicatrization atelectasis, architectural distortion, and traction bronchiectasis or bronchiolectasis (Figures 4A, 4B, 5A, 5D, 5F).In addition, signs of pulmonary hyperinflation with paracicatricial emphysema, hyperlucent areas associated with poor vascularization, bullae and signs of increased lung dimensions like diaphragmatic depression and tracheal dilation are also detected (Figures 4D, 4E, 4F, 5D, 5E). A tendency to the decrease of ground-glass opacities, consolidations, as well as cavitating and the tree-in-bud lesions, has been observed. On the contrary, a predisposition to the raise of the diffuse emphysema, bullae, and traction bronchiectasis is also found. 

### Differentiation between active and residual lesions

PCM patients appropriately treated sometimes present respiratory complaints again, and three possibilities should be considered: relapse, with active disease; clinical manifestations of pulmonary sequelae; and other disease, such as influenza or pneumonia. In this situation, the differential diagnosis between active PCM and its residual form should be performed. On chest CT scan, the presence of ground-glass opacities does not help to differentiate active from residual lesions, because they appear in both stages. On the other hand, tree-in-bud lesions are diagnostic of active disease, meaning bronchial dissemination of the disease (Figure 2E), although this type of lesion is also observed in other infectious diseases. 

### Classification of severity

The active and residual lesions are classified into mild, moderate, and severe according to the pulmonary area involved and the kind of alterations (Table 2). Thus, pulmonary parenchyma was divided into three parts called upper, middle, and lower zones, i.e., upper: above the carina; middle: below the carina and above the inferior pulmonary vein; and lower: below the inferior pulmonary vein [25]. 

 An accurate delimitation is not required, as establishing the extension of the parenchymal involvement and the type of lesions is the goal. Nevertheless, the coronal and sagittal views are valuable for the proposed analysis. 

**Table 2.** Severity of pulmonary involvement in paracoccidioidomycosis patients on high resolution computed tomography scans. Evaluation of active disease and residual forms.

Severity	Active lesions	Residual lesions
**Mild**	One or two focal lesions in consolidation or ground-glass opacities and/or alveolar pure opacities of any size. *Other possible findings*: nodules, bronchial wall thickening.	Only discrete fibrotic lesions: parenchymal bands, traction bronchiectasis, nodules (1-3 cm in diameter with or without calcifications). Normal CT: quite unlikely.
**Moderate**	≥3 focal lesions in consolidation or ground-glass opacities and/or interstitial lesions occupying <⅓ of the total parenchyma area. *Other possible findings*: nodules, bronchial wall thickening, tree-in-bud, bronchiectasis.	Irregular interlobular septal thickening, parenchymal bands, nodules, bronchial wall thickening, focal paracicatricial emphysema, and thin walled cavity-gas-filled space.
**Severe**	Interstitial or mixed lesions occupying >⅓ of the total parenchyma area. *Other possible findings*: nodules, bronchial wall thickening, tree-in-bud, bronchiectasis, cavitary lesions usually in large number.	Bronchial wall thickening, bronchiectasis, honeycombing with signs of emphysema: paracicatricial, bullous, and/or generalized.

## Future directions

Correlations between our imaging severity classification and functional respiratory tests should be evaluated, in order to simplify and to improve the management of PCM patients. These studies are already ongoing.
